# Sweeteners: erythritol, xylitol and cardiovascular risk—friend or foe?

**DOI:** 10.1093/cvr/cvaf091

**Published:** 2025-05-30

**Authors:** Bettina K Wölnerhanssen, Anne Christin Meyer-Gerspach, Arduino Arduini, Angelo D’Alessandro, Edoardo Gronda, Stefano Carugo, Mario Bonomini, Maurizio Gallieni, Valentina Masola, Anne Angelillo-Scherrer, Tommaso Prosdocimi, Gary D Lopaschuk

**Affiliations:** St. Clara Research Ltd at St. Claraspital, Kleinriehenstrasse 43, Basel CH-4058, Switzerland; Faculty of Medicine, University of Basel, Basel 4001, Switzerland; St. Clara Research Ltd at St. Claraspital, Kleinriehenstrasse 43, Basel CH-4058, Switzerland; Faculty of Medicine, University of Basel, Basel 4001, Switzerland; Department of Research and Development, Iperboreal Pharma Srl, Pescara 65121, Italy; Research Department, CoreQuest Sagl, Lugano 6900, Switzerland; Department of Biochemistry and Molecular Genetics, University of Colorado Anschutz Medical Campus, Aurora, CO 80045, USA; IRCCS Ca' Granda Foundation, Cardio Renal Program, UOC Nephrology, Dialysis and Adult Renal Transplant Program, Ospedale Maggiore Policlinico, Milan 20122, Italy; IRCCS Ca' Granda Foundation, Department of Cardio-Thoracic-Vascular Diseases, Ospedale Maggiore Policlinico, Milan 20122, Italy; Department of Clinical Sciences and Community Health, University of Milan, Milan 20122, Italy; Department of Medicine and Aging Sciences, G. d’Annunzio University, Chieti 66100, Italy; Department of Biomedical and Clinical Sciences, University of Milano, Milano 20157, Italy; Department of Biomedical Sciences, University of Padova, Padova 35121, Italy; Department of Hematology and Central Hematology Laboratory, Inselspital, Bern University Hospital, University of Bern, Bern 3010, Switzerland; Department for BioMedical Research, University of Bern, Bern 3008, Switzerland; Department of Research and Development, Iperboreal Pharma Srl, Pescara 65121, Italy; Cardiovascular Research Centre, University of Alberta, Edmonton, AB, Canada

**Keywords:** Sugar alcohols, Xylitol, Erythritol, Thrombocyte aggregation, Vascular health

## Abstract

Hyperglycaemia harms vascular health and promotes platelet aggregation. Reducing glucose concentration is crucial, and sugar alcohols may aid this effort. Used for over 50 years in food, cosmetic, and pharmaceutical industries, erythritol and xylitol minimally affect plasma glucose and insulin levels while promoting the release of beneficial gastrointestinal hormones such as e.g. glucagon-like peptide-1. These properties make them particularly appealing for individuals with diabetes, obesity, and metabolic syndrome. Recent pilot trials suggest that xylitol and erythritol might temporarily alter platelet aggregation. Studies on critically ill patients receiving large intravenous doses and Mendelian randomisation trials do not link sugar alcohols to significant cardiovascular risks. Sugar alcohols are also endogenously produced in the body, and while their increased production under certain conditions is not fully understood, it requires further research. This review discusses the physiology and metabolism of erythritol and xylitol, and other sugar alcohols, their roles in metabolomic profiling, effects on platelet aggregation and cardiovascular risk, related genetic disorders, vascular impacts, and usage in critically ill patients.

**Time of primary review: 30 days**

## 1. Introduction

Sugar alcohols, particularly erythritol and xylitol, have been implicated in increased cardiovascular (CV) risk, although a direct causal relationship between their use and CV events is lacking. Given their widespread use in food products and pharmaceutical formulations, it is crucial to comprehensively evaluate the existing data. This review explores the physiology and metabolism of erythritol and xylitol, and other sugar alcohols, their roles in metabolomic profiling, their effects on platelet aggregation and CV risk, associated genetic disorders, vascular impacts, and their application in critically ill patients.

## 2. Physiology and metabolism of erythritol and xylitol

Erythritol and xylitol are naturally occurring sugar alcohols found in berries and vegetables, with broad applications in the food, pharmaceutical, and cosmetic industries.^1–3^ Upon ingestion, erythritol is rapidly absorbed in the small intestine, with most excreted unchanged in the urine and a small portion metabolized to erythronate.^4^ The absorption rate is dose-dependent, as demonstrated in a recent trial where higher doses (50 g) resulted in slower absorption compared with lower doses (10 g, 25 g), suggesting a saturable process.^4^ Ingestion of erythritol results in elevated plasma erythritol concentrations, with maximum levels (Cmax) reached within 30–60 min: Cmax 1810.6 ± 124.6 μM (10 g), 3676.9 ± 251.2 μM (25 g), and 5404.3 ± 450.6 μM (50 g).^4^ Similarly, Hootman *et al.*^5^ reported that ingesting 50 g of erythritol raised plasma levels to ∼5500 μM within 50 min.^5^ In a recent trial ingestion of 30 g of erythritol resulted in plasma concentrations peaking at 7680 μM after 30 min.^6^ In this trial, plasma erythritol concentrations were measured up to 7 days post-ingestion, with elevated concentrations persisting for up to 2 days compared with baseline.^6^

In contrast, xylitol is only absorbed 50% and mainly metabolized in the liver, while the non-absorbed fraction enters the colon. This partial absorption explains why rapid ingestion of large amounts of xylitol can lead to osmotic diarrhoea. Following a 30 g dose of xylitol, plasma xylitol concentrations were found to peak at ca. 630 µM approximately 30 min post-ingestion return to baseline within 4–6 h.^7^ Absorption studies have so far been conducted exclusively in healthy individuals. However, in people with obesity or diabetes, altered digestive, metabolic, or renal functions could influence how these sugar alcohols are absorbed, metabolized, and excreted. In the colon, xylitol is fermented by gut microbiota and studies with human faecal cultures suggest xylitol might act as a prebiotic, increasing the formation of short-chain fatty acids such as butyrate.^8,9^ The absorbed part of xylitol is further metabolized mainly in the liver within the non-oxidative branch of the pentose monophosphate shunt to produce first D-xylulose and then D-xylulose 5-phosphate (Xyl-5-P), the latter being rapidly converted to glycolytic intermediates.^10^ While consumption of erythritol has no impact on plasma glucose and insulin levels, xylitol leads to a small increase.^11^

A long-term feeding trial on xylitol was carried out in 1972–1974 in Turku, Finland. In this trial, 125 healthy subjects lived on strict diets over 2 years and received sucrose, fructose, or xylitol *ad libitum* as their only sweetener to study the impact on oral health. Subjects in the xylitol group (*n* = 52) consumed an average of 67 g of xylitol per day, but the highest daily doses of xylitol were 200–400 g per day. A total of 35 subjects in the xylitol group were considered to have consumed exceptionally high quantities of xylitol (100–149 g/d). Medical research teams continuously monitored all participants, and no adverse events were registered besides some dose-dependent gastrointestinal discomfort.^12^

**Figure 1:**
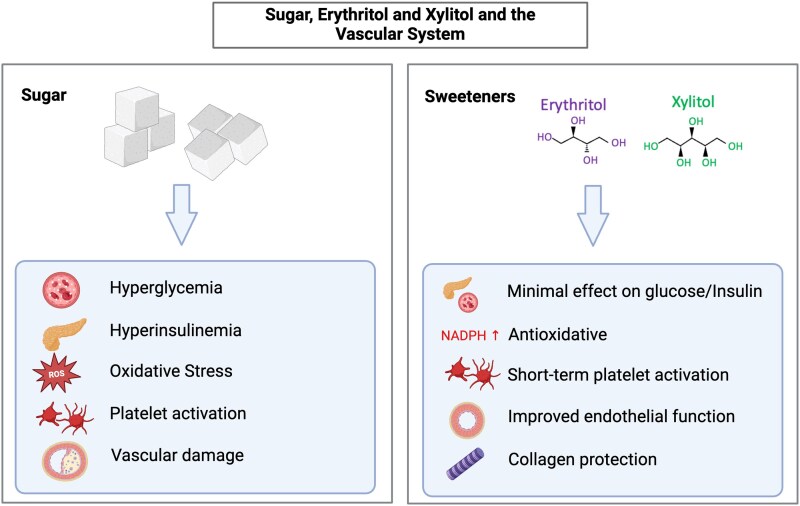
Sugar, erythritol and xylitol and the vascular system. NADPH, nicotinamide adenine dinucleotide phosphate; ROS, reactive oxygen species.

**Figure 2:**
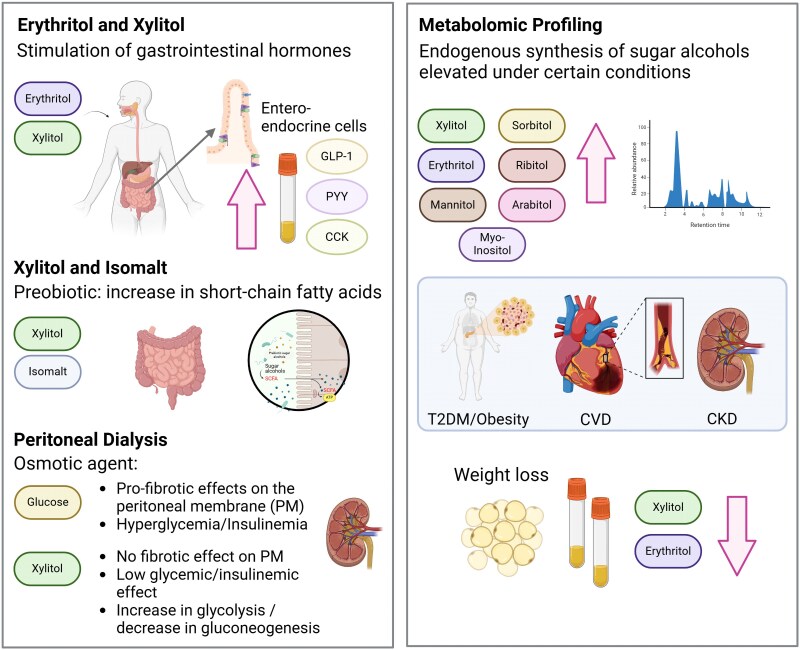
Different effects of sugar alcohols. ATP, adenosine triphosphate; CCK, cholecystokinin; CKD, chronic kidney disease; CVD, cardiovascular disease; GLP-1, glucagon-like peptide-1; PYY, peptide tyrosine tyrosine; SCFA, short chain fatty acids; T2DM, type 2 diabetes mellitus.

More recent studies have repeatedly demonstrated that acute erythritol and xylitol consumption—despite their lack in calories—trigger gastrointestinal satiation hormones (incretins) release (namely cholecystokinin, glucagon-like peptide-1, and peptide tyrosine tyrosine), and erythritol has also been shown to lower concentrations of the hunger hormone ghrelin.^11,13–15^ Downstream effects of gastrointestinal hormones (e.g. a dose-dependent delay in gastric emptying rates in response to erythritol and xylitol intake and activation of brain networks involved in regulating appetite and reward) could also be observed.^11,13,16^ Moreover, a beverage (preload) sweetened with erythritol leads to a significant decrease in subsequent energy intake from an *ad libitum* buffet meal compared with preloads sweetened with sucralose or sucrose or plain water (placebo), which demonstrates the satiating effect of erythritol.^17^ However, data on the long-term effects of daily xylitol or erythritol consumption on caloric intake or body weight are not yet available *Figures 1 and 2*.

## 3. Genetic disorders involving components of the hexose monophosphate shunt

In hereditary metabolic disorders caused by deficiencies in transaldolase (TAL), transketolase (TKL), or sedoheptulokinase (SHK), plasma concentrations of endogenously produced erythritol and other sugar alcohols are chronically elevated:

In TAL-deficiency, patients present with hepatosplenomegaly, congenital heart defects, abnormal liver function, cholestatic jaundice, elevated liver enzymes, hepatic fibrosis or cirrhosis, haemolytic anaemia, thrombocytopenia and abnormal skin (e.g. cutis laxa).^18–20^ TAL-deficiency results in the accumulation of several metabolites (e.g. sedoheptulose, mannoheptulose, sedoheptulose 7-phosphate, erythritol, arabitol, ribitol, sedoheptitol and perseitol, and erythronic acid.^18,20^

In TKL-deficiency, patients present with short stature, developmental delay, congenital heart disease, and cataracts.^21^ TKL-deficiency results in elevated concentrations of several metabolites: Elevated urinary excretion of erythritol, arabitol, ribitol, and pent(ul)ose-5-phosphates, as well as elevated plasma concentrations of erythritol, arabitol, and ribitol.

Isolated deficiency in SHK is a rare, hereditary disorder characterized by high urine levels of sedoheptulose and erythritol, and low-to-normal excretion of sedoheptulose-7P.^22^ Two patients described with SHK-deficiency presented with neonatal cholestasis, hypoglycaemia, respectively, congenital arthrogryposis multiplex, multiple contractures, and dysmorphisms.^22^ Although patients with these genetic conditions exhibit chronically elevated erythritol levels, none appear to increase the risk of blood clot formation.

In pentosuria, a benign and asymptomatic inborn error of metabolism, 1–4 g per day of pentose L-xylulose is excreted in the urine and the plasma concentration of xylulose reaches 80 µM.^23^

Given its structural similarity to xylitol, xylulose ought to exhibit comparable chemical actions, potentially enhancing stimulus-induced platelet aggregation in response to multiple agonists (e.g. ADP, thrombin, collagen) and promoting thrombus formation *in vivo—*unless the supposed ‘pharmacological’ effect of xylitol on platelet aggregation is extremely specific. The studies by Witkowski *et al.*^7^ were conducted at a xylitol concentration almost three times lower (30 µM) than the plasma concentration of xylulose in pentosuric subjects. Interestingly, despite the elevated xylulose levels, pentosuria is harmless and not associated with health problems.

## 4. Sugar alcohols in metabolomic profiling studies

Metabolites are molecules formed as by-products or end-products of metabolic processes, potentially serving as biomarkers for health and disease. Apart from the exogenous supply through natural sources (e.g. berries, mushrooms, cauliflower) and by use as a sweetener, erythritol and xylitol are also endogenously produced by the human body (=metabolites)—as are other sugar alcohols (e.g. mannitol, sorbitol, ribitol).^5,10^ Approximately 5–15 g of xylitol is formed endogenously in the human body daily.^24^ Under certain conditions, endogenous production of sugar alcohols increases. The reason why the body produces more sugar alcohols in these cases is still largely unexplored. Recent studies by Witkowski *et al.*^7^ suggested that higher fasting plasma concentrations of the two metabolites erythritol and xylitol are associated with increased CV risk and might predict major adverse cardiovascular events (MACE).^6,7^ Unfortunately, as in many other metabolomic profiling studies, dietary intake of the two substances was not assessed and therefore the source of the two substances (endogenous vs. exogenous) remains unclear. In a trial published by Wang *et al*.^25^ in 2019, serum samples were prospectively collected from >3500 patients in 1987–1989 and elevated erythritol levels were found to be associated with increased risk for coronary heart disease.^25^ However, erythritol had only been approved by the FDA for use as a food additive in the US in 2001, and therefore, erythritol found in these blood samples must have been from endogenous synthesis.

In the recent study by Witkowski *et al*.,^6^ where plasma erythritol levels were associated with increased MACE risk, the majority of investigated subjects (‘US validation cohort’) were also enrolled *before* erythritol was used as a sugar substitute and therefore erythritol levels most likely reflect endogenous production. The EU validation cohort enrolled patients more recently (until 2018) when erythritol was used on the EU market. The authors, therefore, speculate that erythritol levels in their EU cohort originated from a combination of ingestion and endogenous production.^6^ However, at the time, erythritol was still a niche product in Europe, with limited consumption, particularly among individuals over 60 years of age. This suggests that the observed findings likely reflect primarily endogenous production. The same authors found also elevated xylitol concentrations to be associated with increased MACE risk.^7^ Xylitol has been used as a sweetener in the US and Europe since the 1960 / 1970's. However, as plasma xylitol levels rapidly return to baseline (=fasting) concentrations within hours after ingestion, the fasting plasma levels observed in their observational cohort represent endogenous production and are independent of dietary xylitol intake.^7^ Although derived from the same patient cohorts, the authors separately published findings linking circulating plasma erythritol and xylitol levels to an increased risk of MACE, based on targeted metabolomics analysis.^6,7^ Metabolomic analyses usually yield an abundance of metabolites, and clusters of metabolites (in this case, sugar alcohols) are described to highlight patterns and relationships. In their initial publication, untargeted metabolomics revealed that elevated plasma concentrations of several sugar alcohols (e.g. arabitol, erythritol, xylitol, myoinositol, threitol) and other metabolites were linked to MACE, consistent with prior studies.^6^ However, the focus was primarily on erythritol and xylitol, given their dual role as metabolites and sweeteners. This raises the question of whether erythritol levels were elevated to the same extent as xylitol and other sugar alcohols (e.g. ribitol, sorbitol, mannitol) or if metabolites from other categories were similarly increased in the same patients studied by Witkowski *et al*.^6^

Previous studies have also found elevated circulating concentrations of erythritol precede coronary heart disease,^25,26^ associated with diabetes,^27,28^ obesity,^5^ and arterial stiffness,^29^ and may therefore be a marker of impaired metabolism. The endogenous production of a variety of sugar alcohols—such as mannitol, sorbitol, lactitol, ribitol, and arabitol—appear up-regulated in other diseases, which could be shown, e.g. in tissue samples of hepato-cellular carcinoma,^30^ and serum samples of stroke patients.^31^ In major trauma patients, a metabolomic signature for patients with a prolonged intensive care unit (ICU) stay revealed that—amongst other metabolites—increased xylitol levels seemed of predictive value.^32^ Also, in a large epidemiological study with over 11’000 subjects using data from the EPIC-Norfolk cohort, the metabolites erythritol, erythronate, ribitol, arabitol, xylitol, and myo-inositol were found to be associated with an increased hazard ratio for several diseases (e.g. renal disease, heart failure, peripheral artery disease, ischaemic heart disease, and stroke).^33^

Interestingly, some studies report a decrease in plasma sugar alcohol levels in patients after a weight-loss program. From the POUNDS lost trial—a weight loss program in overweight and obese but normoglycemic patients who were followed over 2 years—higher levels of erythritol, its metabolite erythronate as well as other sugar alcohols, such as mannitol, sorbitol, myoinositol, arabitol and xylitol, were related to a higher atherosclerotic cardiovascular disease (ASCVD) risk.^34,35^ Elevated erythritol levels were also found to be related to insulin resistance (IR). Weight loss led to a decrease in plasma erythritol concentrations, and the authors found a significant association between changes in erythritol and a reduction in fasting insulin. More pronounced decreases in erythritol in response to the weight loss intervention were also significantly related to greater reductions in ASCVD risk estimates. The 6-month decrease in erythritol was associated with long-term (2-year) improvements in atherogenic lipids.^34,35^ Also, in another weight-loss program—the randomized controlled Diabetes Remission Clinical Trial (DiRECT)—participants in the intervention group were found to have reduced levels of erythronate and related metabolites (such as ribitol and erythritol).^36^ Lastly, metabolite profiling of obese individuals before and after a 1-year weight loss program revealed that lower baseline levels of xylitol predicted a more significant decrease in body weight and erythritol levels decreased in response to weight loss.^37^

Overall, the traditional limitation of all these observational clinical studies is that, by design, they can only show association, not causation. In addition, unmodelled confounding variables (e.g. diet) may have influenced their results (directly or indirectly) by factors not included in their models. Moreover, although factors like diet, medications, and the gut microbiome can influence metabolite levels, the metabolome is highly heritable and significantly shaped by genetic factors.^38–40^ Recent genome-wide association studies have identified variants strongly associated with circulating erythritol concentrations, including variants near TKT (encoding TKL) and AKR1A1 (encoding Aldo-Keto Reductase Family 1 Member A1).^39,40^ Based on these observations, Khafagy *et al.*^38^ carried out bidirectional Mendelian randomisation to investigate the potential causal associations between circulating plasma erythritol and associated cardiometabolic disease.^38^ Mendelian randomisation analyses did not find evidence for a causal association between circulating erythritol and coronary artery disease, diabetes, fasting glucose, eGFR, or chronic kidney disease, nor was any evidence found that the evaluated cardiometabolic and anthropometric traits significantly influenced erythritol concentration. Rather, Mendelian randomisation indicated erythritol may decrease BMI.^38^

Why endogenous production of sugar alcohols is found in certain diseases remains unclear. One suggestive possible explanation would be hyperglycaemia, which is common in people who are overweight or have diabetes, as well as in people with a high intake of sugar. Chronic hyperglycaemia can trigger several additional glucose-utilizing pathways, including the polyol, hexosamine, and pentose monophosphate shunt pathways.^41,42^ In mice, increased urine secretion of erythritol is found when they are fed a sucrose diet over several weeks.^43^ An upregulation of these alternative metabolic pathways might—in theory—explain the association of increased levels of erythritol and xylitol with MACE, as hyperglycaemia and diabetes are known risk factors for MACE. However, in the studies by Witkowski *et al.*,^6^ analyses were adjusted for age, sex, BMI, presence of diabetes, systolic blood pressure, current smoking status, and blood lipids.^6^ The authors report that the association between higher erythritol levels and MACE risk was independent of whether the cohorts were stratified into patients with high or low blood glucose or with/without diabetes.^6^ Also, in their Mendelian randomisation trial, Khafagy *et al.*^38^ could not find evidence that erythritol increases fasting glucose and type II diabetes, nor did they find evidence that cardiometabolic traits significantly influence erythritol concentration.^38^

Recent *in vitro* trials on cardiomyocytes suggest that oxidative stress leads to an overproduction of sugar alcohols such as sorbitol, L-arabitol, xylitol, or dulcitol.^44^ Interestingly, several sugar alcohols appear to possess antioxidant properties.^45^ One may wonder whether the increased production of sugar alcohols in connection with hyperglycaemia is a kind of rescue attempt and serves to both increase utilisation of glucose and to counteract oxidative stress. With the increasing consumption of erythritol and xylitol among individuals at higher cardiometabolic risk (e.g. those with obesity and/or diabetes) in recent years, future observational studies should also consider the potential for reverse causality.

While elevated circulating plasma concentrations of erythritol, xylitol, and other sugar alcohols are associated with various diseases, the underlying cause remains unclear. Future studies should definitely take into account the participants' dietary habits (i.e. consumption of erythritol and xylitol as sweeteners but also intake from natural sources).

## 5. Impact of sugar alcohol consumption on gut microbiota composition and function

The potential impact of sweeteners on gut microbiota and their implications for metabolism, especially glucose tolerance, has attracted considerable attention recently. However, human intervention studies on this topic are limited. Evidence suggests that artificial non-caloric sweeteners like sucralose and saccharin may negatively impact gut microbiota composition and potentially promote metabolic diseases (e.g. impaired glycemic control), rather than preventing them.^46,47^ In contrast, human intervention studies on sugar alcohols have indicated that isomalt, lactitol, maltitol, and xylitol may positively influence gut microbiota composition.^48–50^*In vitro* and *ex vivo* studies using gut microbiota from healthy donors also suggest that some sugar alcohols exhibit prebiotic properties. A recent *ex vivo* study on the functional role of various sweeteners found significant metaproteomic alterations with xylitol, isomalt, maltitol, lactitol, sorbitol, and mannitol, while erythritol did not induce such changes.^51^*In vitro* studies have shown that isomalt is metabolized by bifidobacteria strains, leading to high butyrate concentrations.^48^ Various *in vitro* and *ex vivo* studies have demonstrated that xylitol also increases butyrate and propionate production.^8,9,52^ Notably, the butyrate production with xylitol is significantly higher compared with common prebiotics such as fructo-oligo-saccharides and galacto-oligo-saccharides.^8,9^ Short-chain fatty acids like butyrate and propionate are beneficial in that they provide energy to the colonic epithelial cells, and play an important role in maintaining gut immunity, and in supporting gut barrier function.^53^ A recent *ex vivo* study using a dynamic simulator of the colonic microbiota inoculated with pooled faecal samples from children found that xylitol dose-dependently increased the abundance of Lachnospiraceae, particularly Blautia, Anaerostipes, and Roseburia, and enhanced butyrate production, improving epithelial integrity in Caco-2 cells.^52^ The species Blautia wexlerae has been linked to potential benefits in reducing obesity and type II diabetes.^54,55^

While a significant proportion of xylitol enters the colon, erythritol is mostly absorbed in the small intestine.^4^ Moreover, erythritol, which is only minimally available to the colon, appears non-fermentable, as shown in studies with fresh human faecal microbiota.^56^ Overall, xylitol appears to have prebiotic effects, whereas erythritol likely has no impact on the gut microbiota. However, long-term controlled human intervention trials investigating the effects of xylitol and erythritol are still needed.

## 6. Effects of erythritol and xylitol on vascular function

Endothelial dysfunction and arterial stiffness are critical factors in the development of CV disease in individuals with diabetes mellitus. One important contributing factor leading to endothelial dysfunction is hyperglycaemia which promotes oxidative stress.^57^*In vivo*, administration of erythritol prevents endothelial dysfunction in diabetic rats and *in vitro* tests show that erythritol—and other sugar alcohols—are potent hydroxyl radical scavengers.^45^ Extensive *in vitro* studies on human umbilical vein endothelial cells, confirmed that erythritol exerts a number of endothelium-protective properties under hyperglycaemic conditions.^58^ In a pilot trial in patients with diabetes, vascular function in response to acute (2 h after 24 g of oral erythritol) and chronic (36 g/d over 4 weeks) erythritol consumption was examined. This study found that erythritol consumption acutely improves small vessel endothelial function (fingertip peripheral arterial tonometry), and chronic treatment reduced central aortic stiffness (decreased central pulse pressure).^59^ In a randomized controlled trial (RCT) on obese normoglycemic patients, no statistically significant effect of erythritol or xylitol intake on vascular function (left-brachial pulse wave velocity and arteriolar-to-venular diameter ratio) was observed.^60^ The discrepancy in these two *in vivo* human studies may be due to differences in glycemic control: as erythritol seems to act as an antioxidant, an effect might only be seen in hyperglycaemic patients.

In the context of vascular health, it's noteworthy that xylitol appears to impact skin collagen, which may also be relevant for vascular collagen. A 3-month study in healthy and diabetic male rats showed that dietary xylitol (10%) increases hydroxyproline levels and skin thickness in healthy rats.^61^ In diabetic rats, acid-soluble collagen levels, initially lower than in healthy controls, rose significantly with xylitol, while collagenase-soluble levels remains unchanged. Xylitol reduces hexose content and collagen fluorescence, suggesting it influences both collagen synthesis and glycosylation.^61^*In vitro* studies revealed varying protective effects of sugar alcohols, with erythritol being most effective, followed by xylitol, against collagen denaturation by guanidine hydrochloride.^62^ The presence of OH groups in sugar alcohols likely stabilizes collagen by forming additional hydrogen bonds.^62^ Further investigation is needed on the effect of erythritol and xylitol on human vascular collagen.

**Figure 3:**
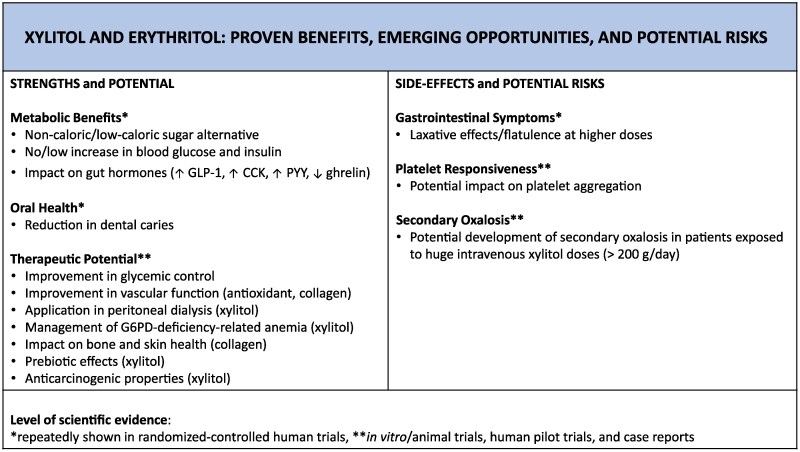
Xylitol and erythritol: proven benefits, emerging opportunities, and potential risks. CCK, cholecystokinin; GLP-1, glucagon-like peptide-1; G6PD, glucose-6-phosphate dehydrogenase; PYY, peptide tyrosine tyrosine.

In the context of vascular health, also the NADPH-generating effect of xylitol should be mentioned. Human erythrocytes contain an NADP-linked xylitol dehydrogenase capable of regenerating NADPH by oxidising xylitol to L-xylulose.^63^ NADPH is critical in preventing damage to cellular structures caused by an oxidative challenge by serving as a substrate for the enzyme glutathione reductase. Reduced glutathione can be used to convert hydrogen peroxide to water and prevent damage to cellular structures, particularly the cell walls of red blood cells (RBCs), which have limited ability to repair themselves once they mature.^64^ Therefore, xylitol can be of pharmaco-medical use to improve glucose-6-phosphate dehydrogenase deficiency anaemia, the most common inborn enzyme disorder in all populations.^65^*In vivo* studies in rabbits have clearly shown that xylitol improves acetylphenylhydrazine-induced haemolytic anaemia.^63,66^ In fact, compared with rabbits injected intraperitoneally with acetylphenylhydrazine alone, the infusion of an isotonic xylitol solution was found to maintain and restore hematological parameters, such as packed cell volume, haemoglobin concentration, reduced glutathione content, reticulocyte count and erythrocyte survival.^66^*Figure 3*.

## 7. Platelet aggregation and CV risk

Various effects associated with high added sugar consumption, such as hypertension, inflammation, weight gain, IR, diabetes, dyslipidemia, and fatty liver disease, contribute to increased CV disease.^67,68^ Hyperglycaemia and IR lead to a prothrombotic state in diabetic patients with changes in platelet numbers and activation, which leads to platelet hypersensitivity, coagulation disorders, and hypofibrinolysis.^69^ Both acute and chronic hyperglycaemia contribute to this procoagulant state in diabetic and non-diabetic subjects.^70,71^ An anti-aggregating treatment with aspirin is often recommended for diabetic patients in secondary prevention of atherothrombotic CV events, but also in primary prevention in the case where at least one additional major risk factor for CVD is present.^72^ However, high plasma glucose levels acutely reduce the anti-aggregating effect of aspirin.^73^ Therefore, patients with diabetes and healthy subjects are advised to limit sugar intake. Sugar replacement with various alternatives can—in part—help to meet this goal. However, questions arise about whether these substances are a better choice concerning CV risk. While an increase in circulating erythritol and xylitol production was found to be associated with an increased risk of a major cardiovascular event (MACE), Witkowski *et al.*^7^ postulated that there might also be direct effects of these substances that lead to increased CV disease.^6,7,74^ In their publications, they presented data from various tests: *in vitro* tests on human thrombocytes exposed to erythritol or xylitol, *in vivo* clot formation in a murine model, and *ex vivo* data from thrombocyte stimulation tests of healthy human subjects consuming xylitol or erythritol. When human platelet-rich plasma from healthy volunteers are exposed to erythritol, platelets showed a dose-dependent increase in aggregation in response to the stimulators ADP and TRAP6.^6^ Erythritol exposure increases intracellular calcium levels and enhances platelet activation markers such as P-selectin expression and GP IIb/IIIa activation. These findings suggest erythritol directly influences platelet activity, potentially contributing to clot formation.^6^ Analogue studies were carried out with xylitol with similar results.^7^ Their findings revealed that exposure to the two substances enhanced thrombin-induced calcium release in platelets. Additionally, platelet activation markers, such as P-selectin expression and glycoprotein activity, were elevated, indicating increased platelet responsiveness.^6,7,74^ However, *in vitro* thrombocyte stimulation tests are of limited significance and cannot be easily transferred to the *in vivo* conditions in the human body. For instance, *in vitro*, acute hyperglycaemia induces platelet hyperreactivity to agonist stimulation by increasing osmolarity, subsequently affecting intracellular signalling in platelets.^75^ It is possible that high concentrations of xylitol or erythritol may induce platelet hyperreactivity by increasing osmolarity, although the molecule *per se* has no specific thrombogenic effect.

Using a FeCl₃-induced carotid artery injury model in mice, Witkowski *et al.*^6^ observed that elevated erythritol levels significantly increase the speed of clot formation and reduce the time until blood flow ceased.^6^ However, in this murine model, erythritol was administered intravenously, which might have led to an osmotic stimulation of thrombocyte aggregation and impacted clot formation. In the case of xylitol, the authors had to inject xylitol intraperitoneally as it was not absorbed enough in the mice's intestines when given orally.^7^ Detailed long-term feeding trials (up to 2 years) in rats and mice with both erythritol and xylitol (using diets containing up to 20% of these substances) did not report abnormalities in CV events and no reduction in lifespan.^76–78^

In separate parallel pilot trials authored by Witkowski *et al.*,^7^ 10 healthy human subjects were given either 30 g of xylitol, or 30 g erythritol, or 30 g glucose *per os*, and the blood platelet response to *ex vivo* stimulation with TRAP6 and ADP was examined at baseline and once after 30 min.^7,74^ In these trials, increased platelet responsiveness was observed following xylitol and erythritol ingestion, while in contrast, glucose had no effect. Previous studies demonstrate that hyperglycaemia induces platelet activation in healthy and diabetic individuals. However, higher blood glucose levels or chronically elevated glucose levels are probably needed.^70,71^ With 10 subjects in each group, the studies on humans were small; there was no placebo arm, and only a single time point after ingestion was investigated (after 30 min). Also, the parallel design does not allow to directly comparing the effects of the three sweeteners in the same subject. It should be noted that environmental influences affect short-term platelet responsiveness, e.g. acute physical activity can increase platelet aggregation and the tendency to aggregate can be reduced or increased post-prandially in response to various foods.^79–83^ While these findings by Witkowski *et al.*^74^ warrant further research, at this point it is not clear whether the described effect can be replicated in larger placebo-controlled randomized studies, whether the effect is also found *in vivo* (rather than *ex vivo*), whether it is only a short-term phenomenon (as with other post-prandial observations), whether the observations are of clinical significance, and which underlying mechanisms might explain the findings. According to the NCT registry, two independent research groups are currently investigating the effects of erythritol and/or xylitol on human platelet responsiveness *in vivo* (NCT 05967741 and NCT 04966299).

Whether other sugar alcohols have any effects on platelet responsiveness remains to be determined. Mannitol, which can be used in the treatment of raised intracranial pressure, has been studied in detail and does not seem to affect platelet function: patients undergoing elective craniotomy due to a brain tumour with elevated intracranial pressure received 1 g/kg mannitol within 30 min intravenously, and no clinically relevant changes in platelet function were found after 60 min.^84^ Similarly, mannitol is a common component in almost all currently licenced storage additives for packed RBC products, which are routinely transfused to coagulopathic patients, including massively transfused critically ill patients or chronically transfused patients with hemoglobinopathies such as sickle cell disease, in which hypercoagulability is a common comorbidity.^85^ Furthermore, research has shown that an extract of alditols and monosaccharides (AME) derived from sorghum vinegar, a traditional Chinese medicine, can effectively inhibit multiple stages in the process of platelet aggregation.^86^ AME demonstrates strong inhibition of cyclooxygenase-1 (COX1) and thromboxane A2 synthase (TXS), as well as reduction of thromboxane A2 (TXA2) production. Computational docking studies suggest that alditols like erythritol and xylitol, but also arabitol and sorbitol can directly bind to the active site of COX, indicating a potential anti-thrombotic effect of these substances.^86^

## 8. Diabetes and obesity

In both diabetic and non-diabetic animal models the favourable effects of oral xylitol and erythritol supplementation on glucose and lipid homeostasis have repeatedly been observed.^87–89^ Investigating the mechanisms behind the antihyperglycemic effects, several mechanisms could be found: a dose-dependent inhibition of alpha-amylase and alpha-glucosidase, decreased small intestinal glucose absorption, increased muscle glucose uptake, and significantly delayed gastric emptying.^90,91^ In humans with or without obesity, both acute erythritol and xylitol ingestion also delays gastric emptying.^11^ However, in patients with obesity, daily intake of 36 g erythritol or 24 g xylitol over 5–7 weeks had no impact on small intestinal glucose absorption assessed by means of 3-*Ortho*-methyl-glucose.^92^ Whether the inhibition of alpha-amylase and alpha-glucosidase and/or increased muscle glucose uptake also play a role in the human body, has not been examined so far.

## 9. Beyond sweetness: use of xylitol instead of glucose in critically ill patients

A key component of the increased CVD risk in diabetic and non-diabetic patients is selective IR.^93–97^ The selectivity of IR is based on the observation that not all insulin signalling pathways are affected by IR. As a result, the pathways that still respond normally to insulin may become overstimulated due to the compensatory increase in insulin levels.^98^ One of the first and well-established evidence of such differential loss of responsiveness originated from studies conducted in endothelial cells on the pro-atherogenic and anti-atherogenic insulin signalling arms, Ras/Raf-MAPK-ERK and IRS-PI3K-Akt, respectively.^99–101^ The differential decrease in insulin responsiveness by the two main branches of insulin signalling causes an imbalance in the effects of insulin on the endothelium, leading to the development of a pro-atherogenic profile.

IR can result directly from hyperinsulinemia due to the downregulation of the plasma membrane insulin receptor following chronic exposure to elevated circulating concentrations of the hormone.^102,103^ This tends to happen after significant and prolonged exposure to high insulin levels, especially when induced through medication or continuous parenteral glucose infusion, which is the most powerful insulin secretagogue. Hyperglycaemia is associated with adverse clinical outcomes in critically ill patients regardless of diabetes status.^104–106^ Proposed causes of stress-induced hyperglycaemia include excessive counterregulatory hormones (e.g. corticosteroids, glucagon, growth hormone, catecholamines) and release of the cytokines tumour necrosis factor (TNF)-alpha and interleukin (IL)-1. These factors can promote a transient state of IR, leading to reduced insulin action to suppress gluconeogenesis and reduced insulin-mediated skeletal muscle glucose uptake.^105^ Factors contributing to hyperglycaemia in hospitalized patients include medications (e.g. steroids, catecholamines), parenteral nutrition and intravenous drugs diluted in dextrose. To mitigate this hyperglycaemic condition in critically ill patients, xylitol can be used as a sugar substitute, either as a stand-alone ingredient or as a component of infusional products for clinical parenteral nutrition.^107^ The low glycemic and low insulinemic indices of xylitol are highly desirable effects in critically ill patients,^11^ where hyperglycaemia and/or insulin-resistant states are associated with increased mortality. In addition, limiting renal exposure to glucose is particularly important in diabetic patients. Glucose in the glomerular filtrate is reabsorbed in the proximal tubule by the combined action of the sodium-glucose cotransporters (SGLT) 2 and 1. The synergy of both SGLTs is responsible for a maximum reabsorption capacity of ∼2.5 mol (450 g) of filtered glucose per day.^108^ Since glucose reabsorption is coupled to sodium reabsorption via an energy-driven process, it explains why the kidney is a primary target of diabetes^109^ and why total body sodium concentration is affected, driving hypertension in most diabetics.^110^

The BfArM in Germany (Bundesinstitut für Arzneimittel und Medizinprodukte, the largest drug regulatory authority in Europe) and the Pmda in Japan (Pharmaceuticals and Medical Devices Agency) have approved a maximum intravenous dose of xylitol of 3 g/kg/day and 100 g/day, respectively. In a parallel trial, Schneider *et al.*^111^ treated 55 ICU patients with a complete parenteral nutrition solution containing 50 g of xylitol and 100 g glucose per 1000 mL.^111^ In this group, a maximum of 120 g xylitol was given per day. The control group comprised 56 patients who received complete parenteral nutrition without xylitol, containing 150 g glucose/1000 mL. In the xylitol-group, serum levels of xylitol were measured and reached 3.4 mg/dL (range 0.00–7.84 mg/dL). Twenty per cent of the patients receiving xylitol had stable glucose levels and did not need insulin compared with only 7.1% of the controls. Patients receiving xylitol required 32.6% less insulin than the control group, despite receiving a comparable amount of energy (xylitol: 810.1 kcal/d; controls: 789.8 kcal/d). In this trial, no adverse effects attributable to parenteral nutrition containing xylitol were observed. Furthermore, there was no difference with regard to adverse events between the two patient groups.

In the literature, the only harmful side effect reported after intravenous xylitol administration, though extremely rare, is severe secondary oxalosis. This has been observed following the infusion of very high doses of xylitol above 300 g/day. A recent case in Japan described a patient who received a maximum dose of 500 g/day, with a cumulative total of 2925 g over eight days.^112^ Unfortunately, in this and a few other case reports of severe secondary oxalosis, no details were provided regarding the potential presence of a loss-of-function mutation in genes involved in primary hyperoxaluria in these patients; a congenital autosomal recessive disorder associated with liver enzyme deficiency leading to massive cortical nephrocalcinosis and subsequent renal failure.^113^

Xylitol is currently also in clinical development as an osmo-metabolic agent for treating kidney failure patients on peritoneal dialysis (PD), a home-based renal replacement therapy alternative to haemodialysis.^114–116^ In addition, a xylitol-based peritoneal ultrafiltration solution is in Phase II/III clinical development for treating congestive heart failure patients refractory to diuretic therapy.^117^ The basic concept is to replace most of the glucose used as an osmotic agent in PD therapy with two more biocompatible osmotic agents, xylitol and carnitine, since glucose has long-term pro-fibrotic effects on the peritoneal membrane, which can ultimately lead to ultrafiltration failure of the peritoneal membrane, forcing the patient to abandon this dialysis modality.^118,119^ In addition to this undesirable local effect, glucose absorption from the dialysate further aggravates the already compromised metabolic state, as diabetes is a common co-morbidity in patients with renal failure.^98^

Therefore, glucose's local and systemic effects exacerbate the CV risk in these patients. Replacing glucose in PD therapy with xylitol would limit the hyperinsulinemic state and, consequently, the extent of a pro-atherogenic selective IR condition.^93–98^ Moreover, overexposing the liver to xylitol may also lead to an insulin-independent improvement of glycemic control since a crucial intermediate in the metabolism of liver xylitol is D-xylulose-5-phosphate (Xyl-5-P).^10^ This compound influences the concentration of fructose 2,6-bisphosphate (Fru-2,6-P_2_), a potent phosphofructokinase activator and, therefore, glycolysis.^120^ The synthesis and degradation of Fru-2,6-P_2_ are catalysed by the homodimeric bifunctional enzyme PFK-2 (6-phosphofructo-2-kinase)/FBPase-2 (fructose-2,6-bisphosphatase), which is in turn is regulated by a cAMP-dependent protein kinase and a protein phosphatase 2A.^121^ Activation of the kinase stimulates gluconeogenesis by decreasing Fru-2,6-P_2_, while phosphatase activation stimulates glycolysis by increasing Fru-2,6-P_2_. Since Xyl-5-P is an allosteric activator of protein phosphatase 2A, this leads to a rapid increase in Fru-2,6-P_2_, activation of phosphofructokinase, thereby activating glycolysis and inhibiting gluconeogenesis.^122^ Consistently, the infusion of xylitol during a combination of a pancreatic and hyperglycaemic clamp leads to a significant increase in Xyl-5-P.^123^ Moreover, xylitol also causes a notable decrease in phosphoenolpyruvic carboxykinase mRNA in the liver, suggesting that Xyl-5-P may suppress gluconeogenesis through a direct transcriptional effect.^123^ Xylitol was investigated as an osmotic agent replacing standard glucose-based PD solution in 6 insulin-dependent diabetic patients on PD therapy.^124^ The therapeutic regimen, administered over a median duration of 8.7 months (range: 5–11 months), consisted of three daily exchanges of PD solution containing 1.5% xylitol and one exchange containing 3% xylitol. The total daily dose of xylitol delivered via the peritoneum was 150 g. Serum xylitol concentrations, measured after hypertonic exchange (3% xylitol), peaked at 86 ± 21 mg/dL (5.4 mM) 1-h post-exchange and decreasing sharply to 12 ± 5 mg/dL (0.8 mM) 3 h later. Using the 1.5% xylitol solution, serum xylitol concentrations reached lower peak levels of 35 ± 21 mg/dL (2.3 mM). Notably, these concentrations are significantly higher than the plasma concentrations reported by Witkowski *et al.*^7^ following ingestion of 30 g xylitol (0.629 mM) or used in their *in vitro* experiments (0.030 mM).^7^ They also exceed the plasma concentrations observed by Schneider *et al.*,^111^ who reported levels of 3.4 mg/dL (0.233 mM) after administering 120 g of xylitol parenterally over 24 h.^111^ The nearly 10-fold difference in plasma xylitol concentrations between Bazzato's study and Schneider's findings likely reflects differences in patient renal function.^111,124^ The patients in Bazzato's study had no remaining kidney function, whereas those in Schneider's study retained renal clearance, which can efficiently eliminate excess xylitol. Additionally, the mode of administration differed: Bazzato's patients received xylitol-based PD solutions as boluses into the peritoneal cavity, while in Schneider's study, the xylitol-based solution was administered intravenously over an extended period rather than as a bolus. After 5 months of xylitol-containing PD treatment, patients in Bazzato's study showed a significant decrease in plasma triglycerides, total cholesterol, and inorganic phosphate levels compared with PD solution containing glucose alone. The exogenous insulin requirement was also reduced by up to 50%.^124^ Still, it was also associated with a significant improvement in glycemic control: the percentage of glycated haemoglobin (%HbA1C) after 5 months of treatment with a stand-alone xylitol-based PD solution decreased from 12.9 ± 0.82% to 10.7 ± 1.08%. This antidiabetic action of xylitol-based PD treatment may be explained by the anti-gluconeogenic effect of the Xyl-5-P derived from liver exposure to peritoneal administration of xylitol.

*In vitro* data from various mesothelial and endothelial cell models has consistently shown that xylitol-based PD solutions have no pro-inflammatory, pro-angiogenic or pro-fibrotic effects compared with glucose-based or glucose-free PD solutions. Instead, they preserve mesothelial and endothelial cells' viability and membrane integrity.^125,126^ Xylitol has even been shown to attenuate renal fibrosis by inhibiting bromodomain-containing protein 4 and its downstream transforming growth factor-β pathway.^127^ In addition, PD patients treated daily with a PD solution containing a ternary combination of xylitol, glucose, and carnitine, where the final daily dose of xylitol was no more than 30 g, experienced no side effects, not even minor ones.^115^ Patients on PD have a high prevalence of CV complications and are at an increased risk of CV mortality. To make matters worse, if traditional CV risk factors such as age, IR, and diabetes mellitus, which are very common in these patients, were added, CV complications and mortality would increase exponentially.^98,128^ All these considerations related to the parenteral route of chronic administration of xylitol in critically ill patients were not addressed in the recent publication discussing a potential link between xylitol and CV risk by Witkowski *et al.*^7^ nor by Beer and Allemann in their accompanying editorial.^7,129^ Interestingly, the latter two authors misquoted a paper on xylitol's ability to enhance the anti-cancer effect of the chemotherapeutic drug 5-fluorouracil both *in vitro* and *in vivo*.^130^ In line with a xylitol intervention even beyond the suggested bitterness in the above editorial, two more recent papers seem to confirm the potential anticancer effects of xylitol.^131,132^

## 10. Summary/conclusion

The consumption of erythritol and xylitol stimulates the release of gastrointestinal satiation hormones while the impact on blood glucose and insulin concentrations remains minimal. These effects are desirable in the context of metabolic and CV health. The harmful effects of chronic overconsumption of conventional sugar are well-documented; hyperglycaemia negatively affects vascular health and promotes thrombocyte aggregation. Reducing glucose load—through dietary modifications or replacing glucose in PD—is important, and sugar alcohols might be helpful in reaching this goal. As a preventive measure, consuming erythritol and xylitol can improve oral health, and partially replacing table sugar with these sweeteners may also help in preventing weight issues and metabolic syndrome.

A potential short-term effect of xylitol and erythritol on *ex vivo* human platelet responsiveness has so far been shown in a small pilot trial. These findings need to be replicated in placebo-controlled studies with larger sample sizes and longer durations than 30 min after ingestion. Furthermore, long-term studies on regular consumption of higher amounts of sugar alcohols are lacking. Long-term RCTs focusing on isolated food items do not exist in nutrition research, as they are not practically feasible. Much of the evidence comes from observational studies, which show associations but cannot establish causality. Short-term studies (lasting weeks to a few months) are more feasible and well-designed interventional trials are needed. The mechanisms behind the upregulation of endogenous sugar alcohol production under certain conditions are not fully understood, and further research is necessary. Mendelian randomisation studies suggest that elevated plasma erythritol levels do not directly contribute to increased CV risk, but further carefully designed interventional trials are still warranted.

A general limitation of this review should be highlighted: while the cited studies originate from all continents, to the best of our knowledge, no comparisons have been conducted across different ethnic groups. Furthermore, as these studies were carried out in adults, the findings cannot be directly extrapolated to children.

## References

[1] Mäkinen KK , Söderling E. A quantitative study of mannitol, sorbitol, xylitol, and xylose in wild berries and commercial fruits. J Food Sci 1980;45:367–371.

[2] de Cock P , Makinen K, Honkala E, Saag M, Kennepohl E, Eapen A. Erythritol is more effective than xylitol and sorbitol in managing oral health endpoints. Int J Dent 2016;2016:9868421. 27635141 10.1155/2016/9868421 PMC5011233

[3] Ahuja V , Macho M, Ewe D, Singh M, Saha S, Saurav K. Biological and pharmacological potential of xylitol: a molecular insight of unique metabolism. Foods 2020;9:1592. 33147854 10.3390/foods9111592 PMC7693686

[4] Bordier V , Teysseire F, Senner F, Schlotterbeck G, Drewe J, Beglinger C, Wolnerhanssen BK, Meyer-Gerspach AC. Absorption and metabolism of the natural sweeteners erythritol and xylitol in humans: a dose-ranging study. Int J Mol Sci 2022;23:9867. 36077269 10.3390/ijms23179867 PMC9456049

[5] Hootman KC , Trezzi J-P, Kraemer L, Burwell LS, Dong X, Guertin KA, Jaeger C, Stover PJ, Hiller K, Cassano PA. Erythritol is a pentose-phosphate pathway metabolite and associated with adiposity gain in young adults. Proc Natl Acad Sci U S A 2017;114:E4233–E4240. 28484010 10.1073/pnas.1620079114 PMC5448202

[6] Witkowski M , Nemet I, Alamri H, Wilcox J, Gupta N, Nimer N, Haghikia A, Li XS, Wu Y, Saha PP, Demuth I, König M, Steinhagen-Thiessen E, Cajka T, Fiehn O, Landmesser U, Tang WHW, Hazen SL. The artificial sweetener erythritol and cardiovascular event risk. Nat Med 2023;29:710–718. 36849732 10.1038/s41591-023-02223-9 PMC10334259

[7] Witkowski M , Nemet I, Li XS, Wilcox J, Ferrell M, Alamri H, Gupta N, Wang Z, Tang WHW, Hazen SL. Xylitol is prothrombotic and associated with cardiovascular risk. Eur Heart J 2024;45:2439–2452. 38842092 10.1093/eurheartj/ehae244 PMC11492277

[8] Sato T , Kusuhara S, Yokoi W, Ito M, Miyazaki K. Prebiotic potential of L-sorbose and xylitol in promoting the growth and metabolic activity of specific butyrate-producing bacteria in human fecal culture. FEMS Microbiol Ecol 2017;93:fiw227. 27810878 10.1093/femsec/fiw227

[9] Makelainen HS , Makivuokko HA, Salminen SJ, Rautonen NE, Ouwehand AC. The effects of polydextrose and xylitol on microbial community and activity in a 4-stage colon simulator. J Food Sci 2007;72:M153–M159. 17995737 10.1111/j.1750-3841.2007.00350.x

[10] Ylikahri R . Metabolic and nutritional aspects of xylitol. Adv Food Res 1979;25:159–180. 391001 10.1016/s0065-2628(08)60237-2

[11] Wölnerhanssen BK , Cajacob L, Keller N, Doody A, Rehfeld JF, Drewe J, Peterli R, Beglinger C, Meyer-Gerspach AC. Gut hormone secretion, gastric emptying, and glycemic responses to erythritol and xylitol in lean and obese subjects. Am J Physiol Endocrinol Metab 2016;310:E1053–E1061. 27117004 10.1152/ajpendo.00037.2016

[12] Mäkinen KK , Scheinin A. Turku sugar studies VI. The administration of the trial and the control of the dietary regimen. Acta Odontol Scand 1975;33:105–127. 10.3109/00016357608997712 795261

[13] Wolnerhanssen BK , Drewe J, Verbeure W, le Roux CW, Dellatorre-Teixeira L, Rehfeld JF, Holst JJ, Hartmann B, Tack J, Peterli R, Beglinger C, Meyer-Gerspach AC. Gastric emptying of solutions containing the natural sweetener erythritol and effects on gut hormone secretion in humans: a pilot dose-ranging study. Diabetes Obes Metab 2021;23:1311–1321. 33565706 10.1111/dom.14342 PMC8247993

[14] Sorrentino ZA , Smith G, Palm L, Motwani K, Butterfield J, Archer C, Henderson R, Heldermon C, Gautam S, Brantly ML. An erythritol-sweetened beverage induces satiety and suppresses ghrelin compared to aspartame in healthy non-obese subjects: a pilot study. Cureus 2020;12:e11409. 33194505 10.7759/cureus.11409 PMC7657312

[15] Teysseire F , Bordier V, Budzinska A, Van Oudenhove L, Weltens N, Beglinger C, Wolnerhanssen BK, Meyer-Gerspach AC. Metabolic effects and safety aspects of acute D-allulose and erythritol administration in healthy subjects. Nutrients 2023;15:458. 36678329 10.3390/nu15020458 PMC9863415

[16] Meyer-Gerspach AC , Wingrove JO, Beglinger C, Rehfeld JF, Le Roux CW, Peterli R, Dupont P, O'Daly O, Van Oudenhove L, Wolnerhanssen BK. Erythritol and xylitol differentially impact brain networks involved in appetite regulation in healthy volunteers. Nutr Neurosci 2022;25:2344–2358. 34404339 10.1080/1028415X.2021.1965787

[17] Teysseire F , Flad E, Bordier V, Budzinska A, Weltens N, Rehfeld JF, Beglinger C, Van Oudenhove L, Wolnerhanssen BK, Meyer-Gerspach AC. Oral erythritol reduces energy intake during a subsequent ad libitum test meal: a randomized, controlled, crossover trial in healthy humans. Nutrients 2022;14:3918. 36235571 10.3390/nu14193918 PMC9571225

[18] Verhoeven NM , Huck JH, Roos B, Struys EA, Salomons GS, Douwes AC, van der Knaap MS, Jakobs C. Transaldolase deficiency: liver cirrhosis associated with a new inborn error in the pentose phosphate pathway. Am J Hum Genet 2001;68:1086–1092. 11283793 10.1086/320108 PMC1226089

[19] Eyaid W , Al Harbi T, Anazi S, Wamelink MM, Jakobs C, Al Salammah M, Al Balwi M, Alfadhel M, Alkuraya FS. Transaldolase deficiency: report of 12 new cases and further delineation of the phenotype. J Inherit Metab Dis 2013;36:997–1004. 23315216 10.1007/s10545-012-9577-8

[20] Stincone A , Prigione A, Cramer T, Wamelink MM, Campbell K, Cheung E, Olin-Sandoval V, Gruning NM, Kruger A, Tauqeer Alam M, Keller MA, Breitenbach M, Brindle KM, Rabinowitz JD, Ralser M. The return of metabolism: biochemistry and physiology of the pentose phosphate pathway. Biol Rev Camb Philos Soc 2015;90:927–963. 25243985 10.1111/brv.12140 PMC4470864

[21] Boyle L , Wamelink MMC, Salomons GS, Roos B, Pop A, Dauber A, Hwa V, Andrew M, Douglas J, Feingold M, Kramer N, Saitta S, Retterer K, Cho MT, Begtrup A, Monaghan KG, Wynn J, Chung WK. Mutations in TKT are the cause of a syndrome including short stature, developmental delay, and congenital heart defects. Am J Hum Genet 2016;98:1235–1242. 27259054 10.1016/j.ajhg.2016.03.030 PMC4908149

[22] Wamelink MM , Ramos RJ, van den Elzen AP, Ruijter GJ, Bonte R, Diogo L, Garcia P, Neves N, Nota B, Haschemi A, Tavares de Almeida I, Salomons GS. First two unrelated cases of isolated sedoheptulokinase deficiency: a benign disorder? J Inherit Metab Dis 2015;38:889–894. 25647543 10.1007/s10545-014-9809-1 PMC4551550

[23] Pierce SB , Spurrell CH, Mandell JB, Lee MK, Zeligson S, Bereman MS, Stray SM, Fokstuen S, MacCoss MJ, Levy-Lahad E, King M-C, Motulsky AG. Garrod's fourth inborn error of metabolism solved by the identification of mutations causing pentosuria. Proc Natl Acad Sci U S A 2011;108:18313–18317. 22042873 10.1073/pnas.1115888108 PMC3215002

[24] Hollmann S , Touster O. Non-Glycolytic Pathways of Metabolism of Glucose. New York: Academic Press; 1964.

[25] Wang Z , Zhu C, Nambi V, Morrison AC, Folsom AR, Ballantyne CM, Boerwinkle E, Yu B. Metabolomic pattern predicts incident coronary heart disease. Arterioscler Thromb Vasc Biol 2019;39:1475–1482. 31092011 10.1161/ATVBAHA.118.312236 PMC6839698

[26] Deidda M , Noto A, Cadeddu Dessalvi C, Andreini D, Andreotti F, Ferrannini E, Latini R, Maggioni AP, Magnoni M, Mercuro G; On Behalf of the Capire Investigators. Why do high-risk patients develop or not develop coronary artery disease? Metabolic insights from the CAPIRE study. Metabolites 2022;12:123. 35208197 10.3390/metabo12020123 PMC8876355

[27] Rebholz CM , Yu B, Zheng Z, Chang P, Tin A, Kottgen A, Wagenknecht LE, Coresh J, Boerwinkle E, Selvin E. Serum metabolomic profile of incident diabetes. Diabetologia 2018;61:1046–1054. 29556673 10.1007/s00125-018-4573-7 PMC5878141

[28] Moon JY , Chai JC, Yu B, Song RJ, Chen GC, Graff M, Daviglus ML, Chan Q, Thyagarajan B, Castaneda SF, Grove ML, Cai J, Xue X, Mossavar-Rahmani Y, Vasan RS, Boerwinkle E, Kaplan RC, Qi Q. Metabolomic signatures of sedentary behavior and cardiometabolic traits in US Hispanics/Latinos: results from HCHS/SOL. Med Sci Sports Exerc 2023;55:1781–1791. 37170952 10.1249/MSS.0000000000003205 PMC10523950

[29] Katakami N , Omori K, Taya N, Arakawa S, Takahara M, Matsuoka TA, Tsugawa H, Furuno M, Bamba T, Fukusaki E, Shimomura I. Plasma metabolites associated with arterial stiffness in patients with type 2 diabetes. Cardiovasc Diabetol 2020;19:75. 32527273 10.1186/s12933-020-01057-w PMC7291560

[30] Ismail IT , Fiehn O, Elfert A, Helal M, Salama I, El-Said H. Sugar alcohols have a key role in pathogenesis of chronic liver disease and hepatocellular carcinoma in whole blood and liver tissues. Cancers (Basel) 2020;12:484. 32092943 10.3390/cancers12020484 PMC7072169

[31] Yadav S , Kumar A, Singh S, Ahmad S, Singh G, Khan AR, Chaurasia RN, Kumar D. NMR based serum metabolomics revealed metabolic signatures associated with oxidative stress and mitochondrial damage in brain stroke. Metab Brain Dis 2024;39:283–294. 38095788 10.1007/s11011-023-01331-2

[32] Acharjee A , Hazeldine J, Bazarova A, Deenadayalu L, Zhang J, Bentley C, Russ D, Lord JM, Gkoutos GV, Young SP, Foster MA. Integration of metabolomic and clinical data improves the prediction of intensive care unit length of stay following major traumatic injury. Metabolites 2021;12:29. 35050151 10.3390/metabo12010029 PMC8780653

[33] Pietzner M , Stewart ID, Raffler J, Khaw KT, Michelotti GA, Kastenmuller G, Wareham NJ, Langenberg C. Plasma metabolites to profile pathways in noncommunicable disease multimorbidity. Nat Med 2021;27:471–479. 33707775 10.1038/s41591-021-01266-0 PMC8127079

[34] Heianza, Y, Xue Q, Rood J, Bray G, Sacks F, Qi L. Abstract: changes in plasma levels of nonnutritive sweetener erythritol are related to two-year changes of insulin sensitivity in response to weight-loss diets—the POUNDS lost trial. Diabetes Care 2023;72(Suppl. 1):48-LB.

[35] Heianza Y , Rood J, Champagne CM, Manson JE, Bray G, Sacks FM, Qi L. ABSTRACT MP28: declines in plasma levels of nonnutritive sweetener erythritol are related to two-year improvements in atherosclerotic cardiovascular disease risk estimates among adults with overweight and obesity. Circulation 2024;149:AMP28.

[36] Corbin LJ , Hughes DA, Bull CJ, Vincent EE, Smith ML, McConnachie A, Messow CM, Welsh P, Taylor R, Lean MEJ, Sattar N, Timpson NJ. The metabolomic signature of weight loss and remission in the Diabetes Remission Clinical Trial (DiRECT). Diabetologia 2024;67:74–87. 37878066 10.1007/s00125-023-06019-x PMC10709482

[37] Geidenstam N , Al-Majdoub M, Ekman M, Spegel P, Ridderstrale M. Metabolite profiling of obese individuals before and after a one year weight loss program. Int J Obes 2017;41:1369–1378. 10.1038/ijo.2017.124 28529327

[38] Khafagy R , Paterson AD, Dash S. Erythritol as a potential causal contributor to cardiometabolic disease: a Mendelian randomization study. Diabetes 2024;73:325–331. 37939167 10.2337/db23-0330

[39] Hysi PG , Mangino M, Christofidou P, Falchi M, Karoly ED, Nihr Bioresource I, Mohney RP, Valdes AM, Spector TD, Menni C. Metabolome genome-wide association study identifies 74 novel genomic regions influencing plasma metabolites levels. Metabolites 2022;12:61. 35050183 10.3390/metabo12010061 PMC8777659

[40] Yin X , Chan LS, Bose D, Jackson AU, VandeHaar P, Locke AE, Fuchsberger C, Stringham HM, Welch R, Yu K, Fernandes Silva L, Service SK, Zhang D, Hector EC, Young E, Ganel L, Das I, Abel H, Erdos MR, Bonnycastle LL, Kuusisto J, Stitziel NO, Hall IM, Wagner GR, Kang J, Morrison J, Burant CF, Collins FS, Ripatti S, Palotie A, Freimer NB, Mohlke KL, Scott LJ, Wen X, Fauman EB, Laakso M, Boehnke M. Genome-wide association studies of metabolites in Finnish men identify disease-relevant loci. Nat Commun 2022;13:1644. 35347128 10.1038/s41467-022-29143-5 PMC8960770

[41] Mizukami H , Osonoi S. Pathogenesis and molecular treatment strategies of diabetic neuropathy collateral glucose-utilizing pathways in diabetic polyneuropathy. Int J Mol Sci 2020;22:94. 33374137 10.3390/ijms22010094 PMC7796340

[42] Paul S , Ali A, Katare R. Molecular complexities underlying the vascular complications of diabetes mellitus—a comprehensive review. J Diabetes Complications 2020;34:107613. 32505477 10.1016/j.jdiacomp.2020.107613

[43] Ortiz SR , Field MS. Sucrose intake elevates erythritol in plasma and urine in male mice. J Nutr 2023;153:1889–1902. 37245661 10.1016/j.tjnut.2023.05.022

[44] Zhu C , Gu H, Jin Y, Wurm D, Freidhof B, Lu Y, Chen QM. Metabolomics of oxidative stress: Nrf2 independent depletion of NAD or increases of sugar alcohols. Toxicol Appl Pharmacol 2022;442:115949. 35227738 10.1016/j.taap.2022.115949

[45] den Hartog GJ , Boots AW, Adam-Perrot A, Brouns F, Verkooijen IW, Weseler AR, Haenen GR, Bast A. Erythritol is a sweet antioxidant. Nutrition 2010;26:449–458. 19632091 10.1016/j.nut.2009.05.004

[46] Suez J , Cohen Y, Valdes-Mas R, Mor U, Dori-Bachash M, Federici S, Zmora N, Leshem A, Heinemann M, Linevsky R, Zur M, Ben-Zeev Brik R, Bukimer A, Eliyahu-Miller S, Metz A, Fischbein R, Sharov O, Malitsky S, Itkin M, Stettner N, Harmelin A, Shapiro H, Stein-Thoeringer CK, Segal E, Elinav E. Personalized microbiome-driven effects of non-nutritive sweeteners on human glucose tolerance. Cell 2022;185:3307–3328.e19. 35987213 10.1016/j.cell.2022.07.016

[47] Dalenberg JR , Patel BP, Denis R, Veldhuizen MG, Nakamura Y, Vinke PC, Luquet S, Small DM. Short-term consumption of sucralose with, but not without, carbohydrate impairs neural and metabolic sensitivity to sugar in humans. Cell Metab 2020;31:493–502.e7. 32130881 10.1016/j.cmet.2020.01.014 PMC7784207

[48] Gostner A , Blaut M, Schaffer V, Kozianowski G, Theis S, Klingeberg M, Dombrowski Y, Martin D, Ehrhardt S, Taras D, Schwiertz A, Kleessen B, Lührs H, Schauber J, Dorbath D, Menzel T, Scheppach W. Effect of isomalt consumption on faecal microflora and colonic metabolism in healthy volunteers. Br J Nutr 2006;95:40–50. 16441915 10.1079/bjn20051589

[49] Finney M , Smullen J, Foster HA, Brokx S, Storey DM. Effects of low doses of lactitol on faecal microflora, pH, short chain fatty acids and gastrointestinal symptomology. Eur J Nutr 2007;46:307–314. 17623227 10.1007/s00394-007-0666-7

[50] Beards E , Tuohy K, Gibson G. A human volunteer study to assess the impact of confectionery sweeteners on the gut microbiota composition. Br J Nutr 2010;104:701–708. 20370946 10.1017/S0007114510001078

[51] Sun Z , Wang W, Li L, Zhang X, Ning Z, Mayne J, Walker K, Stintzi A, Figeys D. Comprehensive assessment of functional effects of commonly used sugar substitute sweeteners on ex vivo human gut microbiome. Microbiol Spectr 2022;10:e0041222. 35695565 10.1128/spectrum.00412-22 PMC9431030

[52] Bellanco A , Celcar S, Martinez-Cuesta MC, Requena T. The food additive xylitol enhances the butyrate formation by the child gut microbiota developed in a dynamic colonic simulator. Food Chem Toxicol 2024;187:114605. 38537869 10.1016/j.fct.2024.114605

[53] Mohamed Elfadil O , Mundi MS, Abdelmagid MG, Patel A, Patel N, Martindale R. Butyrate: more than a short chain fatty acid. Curr Nutr Rep 2023;12:255–262. 36763294 10.1007/s13668-023-00461-4

[54] Park S , Zhang T, Kang S. Fecal microbiota composition, their interactions, and metagenome function in US adults with type 2 diabetes according to enterotypes. Int J Mol Sci 2023;24:9533. 37298483 10.3390/ijms24119533 PMC10253423

[55] Rui W , Li X, Wang L, Tang X, Yang J. Potential applications of *Blautia wexlerae* in the regulation of host metabolism. Probiotics Antimicrob Proteins 2024;16:1866–1874. 38703323 10.1007/s12602-024-10274-8

[56] Arrigoni E , Brouns F, Amado R. Human gut microbiota does not ferment erythritol. Br J Nutr 2005;94:643–646. 16277764 10.1079/bjn20051546

[57] Tabit CE , Chung WB, Hamburg NM, Vita JA. Endothelial dysfunction in diabetes mellitus: molecular mechanisms and clinical implications. Rev Endocr Metab Disord 2010;11:61–74. 20186491 10.1007/s11154-010-9134-4 PMC2882637

[58] Boesten DM , Berger A, de Cock P, Dong H, Hammock BD, den Hartog GJ, Bast A. Multi-targeted mechanisms underlying the endothelial protective effects of the diabetic-safe sweetener erythritol. PLoS One 2013;8:e65741. 23755276 10.1371/journal.pone.0065741 PMC3673924

[59] Flint N , Hamburg NM, Holbrook M, Dorsey PG, LeLeiko RM, Berger A, de Cock P, Bosscher D, Vita JA. Effects of erythritol on endothelial function in patients with type 2 diabetes mellitus: a pilot study. Acta Diabetol 2014;51:513–516. 24366423 10.1007/s00592-013-0534-2 PMC4037362

[60] Bordier V , Teysseire F, Drewe J, Madorin P, Bieri O, Schmidt-Trucksass A, Hanssen H, Beglinger C, Meyer-Gerspach AC, Wolnerhanssen BK. Effects of a 5-week intake of erythritol and xylitol on vascular function, abdominal fat and glucose tolerance in humans with obesity: a pilot trial. BMJ Nutr Prev Health 2023;6:264–272. 10.1136/bmjnph-2023-000764 PMC11009538 38618550

[61] Knuuttila ML , Kuoksa TH, Svanberg MJ, Mattila PT, Karjalainen KM, Kolehmainen E. Effects of dietary xylitol on collagen content and glycosylation in healthy and diabetic rats. Life Sci 2000;67:283–290. 10983872 10.1016/s0024-3205(00)00621-4

[62] Usha R , Ramasami T. Stability of collagen with polyols against guanidine denaturation. Colloids Surf B Biointerfaces 2008;61:39–42. 17720461 10.1016/j.colsurfb.2007.07.005

[63] Luzzatto L , Arese P. Favism and glucose-6-phosphate dehydrogenase deficiency. N Engl J Med 2018;378:60–71. 29298156 10.1056/nejmra1708111

[64] Wang YM , Patterson JH, Van Eys J. The potential use of xylitol in glucose-6-phosphate dehydrogenase deficiency anemia. J Clin Invest 1971;50:1421–1428. 4397414 10.1172/JCI106625 PMC292080

[65] Layton MRM , O’Shaughnessy D, Luzzatto L. Glucose-6-phosphate dehydrogenase deficiency. Curr Paediatr 1995;5:190–194.

[66] Ukab WA , Sato J, Wang YM, van Eys J. Xylitol mediated amelioration of acetylphenylhydrazine-induced hemolysis in rabbits. Metabolism 1981;30:1053–1059. 7289879 10.1016/0026-0495(81)90047-0

[67] Chun S , Choi Y, Chang Y, Cho J, Zhang Y, Rampal S, Zhao D, Ahn J, Suh BS, Pastor-Barriuso R, Lima JAC, Chung EC, Shin H, Guallar E, Ryu S. Sugar-sweetened carbonated beverage consumption and coronary artery calcification in asymptomatic men and women. Am Heart J 2016;177:17–24. 27297845 10.1016/j.ahj.2016.03.018

[68] Yang Q , Zhang Z, Gregg EW, Flanders WD, Merritt R, Hu FB. Added sugar intake and cardiovascular diseases mortality among US adults. JAMA Intern Med 2014;174:516–524. 24493081 10.1001/jamainternmed.2013.13563 PMC10910551

[69] Li X , Weber NC, Cohn DM, Hollmann MW, DeVries JH, Hermanides J, Preckel B. Effects of hyperglycemia and diabetes mellitus on coagulation and hemostasis. J Clin Med 2021;10:2419. 34072487 10.3390/jcm10112419 PMC8199251

[70] Lemkes BA , Hermanides J, Devries JH, Holleman F, Meijers JCM, Hoekstra JBL. Hyperglycemia: a prothrombotic factor? J Thromb Haemost 2010;8:1663–1669. 20492456 10.1111/j.1538-7836.2010.03910.x

[71] Vaidyula VR , Boden G, Rao AK. Platelet and monocyte activation by hyperglycemia and hyperinsulinemia in healthy subjects. Platelets 2006;17:577–585. 17127486 10.1080/09537100600760814

[72] American Diabetes Association Professional Practice Committee . 10. Cardiovascular disease and risk management: standards of care in diabetes—2024. Diabetes Care 2024;47(Suppl. 1):S179–S218. 38078592 10.2337/dc24-S010 PMC10725811

[73] Russo I , Viretto M, Barale C, Mattiello L, Doronzo G, Pagliarino A, Cavalot F, Trovati M, Anfossi G. High glucose inhibits the aspirin-induced activation of the nitric oxide/cGMP/cGMP-dependent protein kinase pathway and does not affect the aspirin-induced inhibition of thromboxane synthesis in human platelets. Diabetes 2012;61:2913–2921. 22837307 10.2337/db12-0040 PMC3478557

[74] Witkowski M , Wilcox J, Province V, Wang Z, Nemet I, Tang WHW, Hazen SL. Ingestion of the non-nutritive sweetener erythritol, but not glucose, enhances platelet reactivity and thrombosis potential in healthy volunteers. Arterioscler Thromb Vasc Biol 2024;44:2136–2141. 39114916 10.1161/ATVBAHA.124.321019 PMC11338701

[75] Sudic D , Razmara M, Forslund M, Ji Q, Hjemdahl P, Li N. High glucose levels enhance platelet activation: involvement of multiple mechanisms. Br J Haematol 2006;133:315–322. 16643434 10.1111/j.1365-2141.2006.06012.x

[76] Lina BA , Bos-Kuijpers MH, Til HP, Bar A. Chronic toxicity and carcinogenicity study of erythritol in rats. Regul Toxicol Pharmacol 1996;24:S264–S279. 8933643 10.1006/rtph.1996.0108

[77] Hunter B , Colley J, Street AE, Heywood R, Prentice DE, Magnusson G. Xylitol Tumorigenicity and Toxicity Study in Long-Term Dietary Administration to Rats (Final Report). Vol. 11–14. Basel, Switzerland: Huntingdon Research Centre, Huntingdon, Cambridgeshire, England F Hoffman La Roche Company, Ltd; 1978. p2250.

[78] Hunter B , Graham C, Heywood R, Prentice DE, Roe FJC, Noakes DN. Tumorigenicity and Carcinogenicity Study with Xylitol in Long-Term Dietary Administration to Mice (Final Report). Vol. 20–23. Basel, Switzerland: Huntingdon Research Centre, Huntingdon, Cambridgeshire, England F Hoffman La Roche Company, Ltd; 1978. p1500.

[79] Gow IF , Dockrell M, Edwards CR, Elder A, Grieve J, Kane G, Padfield PL, Waugh CJ, Williams BC. The sensitivity of human blood platelets to the aggregating agent ADP during different dietary sodium intakes in healthy men. Eur J Clin Pharmacol 1992;43:635–638. 1493845 10.1007/BF02284963

[80] Gow IF , Padfield PL, Reid M, Stewart SE, Edwards CR, Williams BC. High sodium intake increases platelet aggregation in normal females. J Hypertens Suppl 1987;5:S243–S246. 3481816

[81] Ahuja KDK , Thomas GA, Adams MJ, Ball MJ. Postprandial platelet aggregation: effects of different meals and glycemic index. Eur J Clin Nutr 2012;66:722–726. 22434051 10.1038/ejcn.2012.28

[82] Spectre G , Ostenson C-G, Li N, Hjemdahl P. Postprandial platelet activation is related to postprandial plasma insulin rather than glucose in patients with type 2 diabetes. Diabetes 2012;61:2380–2384. 22688337 10.2337/db11-1806 PMC3425422

[83] Kristiansen J , Grove EL, Sjuretharson T, Mohr M, Kristensen SD, Hvas A-M. Acute and subacute effects of strenuous exercise on platelet aggregation, coagulation and fibrinolysis in patients with stable coronary artery disease. Thromb Res 2024;236:220–227. 38484628 10.1016/j.thromres.2024.03.007

[84] Arlt K , Frank P, Flentje M, Eismann H, Hermann EJ, Krauss JK, Al-Afif S, Palmaers T. Effect of mannitol on platelet function during elective craniotomy in adult patients with brain tumor. J Neurosurg Sci 2024;68:447–452. 35380206 10.23736/S0390-5616.22.05678-8

[85] Lagerberg JW , Korsten H, Van Der Meer PF, De Korte D. Prevention of red cell storage lesion: a comparison of five different additive solutions. Blood Transfus 2017;15:456–462. 28488968 10.2450/2017.0371-16 PMC5589708

[86] Li J , Yu G, Fan J. Alditols and monosaccharides from sorghum vinegar can attenuate platelet aggregation by inhibiting cyclooxygenase-1 and thromboxane-A2 synthase. J Ethnopharmacol 2014;155:285–292. 24877847 10.1016/j.jep.2014.05.018

[87] Amo K , Arai H, Uebanso T, Fukaya M, Koganei M, Sasaki H, Yamamoto H, Taketani Y, Takeda E. Effects of xylitol on metabolic parameters and visceral fat accumulation. J Clin Biochem Nutr 2011;49:1–7. 21765599 10.3164/jcbn.10-111 PMC3128359

[88] Islam MS , Indrajit M. Effects of xylitol on blood glucose, glucose tolerance, serum insulin and lipid profile in a type 2 diabetes model of rats. Ann Nutr Metab 2012;61:57–64. 22832597 10.1159/000338440

[89] Kishore P , Kehlenbrink S, Hu M, Zhang K, Gutierrez-Juarez R, Koppaka S, El-Maghrabi MR, Hawkins M. Xylitol prevents NEFA-induced insulin resistance in rats. Diabetologia 2012;55:1808–1812. 22460760 10.1007/s00125-012-2527-z PMC3606878

[90] Chukwuma CI , Islam MS. Effects of xylitol on carbohydrate digesting enzymes activity, intestinal glucose absorption and muscle glucose uptake: a multi-mode study. Food Funct 2015;6:955–962. 25656339 10.1039/c4fo00994k

[91] Chukwuma CI , Mopuri R, Nagiah S, Chuturgoon AA, Islam MS. Erythritol reduces small intestinal glucose absorption, increases muscle glucose uptake, improves glucose metabolic enzymes activities and increases expression of Glut-4 and IRS-1 in type 2 diabetic rats. Eur J Nutr 2018;57:2431–2444. 28770335 10.1007/s00394-017-1516-x

[92] Bordier V , Teysseire F, Schlotterbeck G, Senner F, Beglinger C, Meyer-Gerspach AC, Wolnerhanssen BK. Effect of a chronic intake of the natural sweeteners xylitol and erythritol on glucose absorption in humans with obesity. Nutrients 2021;13:3950. 34836205 10.3390/nu13113950 PMC8618859

[93] Brown MS , Goldstein JL. Selective versus total insulin resistance: a pathogenic paradox. Cell Metab 2008;7:95–96. 18249166 10.1016/j.cmet.2007.12.009

[94] Kubota T , Kubota N, Kadowaki T. Imbalanced insulin actions in obesity and type 2 diabetes: key mouse models of insulin signaling pathway. Cell Metab 2017;25:797–810. 28380373 10.1016/j.cmet.2017.03.004

[95] Williams KJ , Wu X. Imbalanced insulin action in chronic over nutrition: clinical harm, molecular mechanisms, and a way forward. Atherosclerosis 2016;247:225–282. 26967715 10.1016/j.atherosclerosis.2016.02.004

[96] Packer M . Potentiation of insulin signaling contributes to heart failure in type 2 diabetes: a hypothesis supported by both mechanistic studies and clinical trials. JACC Basic Transl Sci 2018;3:415–419. 30062227 10.1016/j.jacbts.2018.04.003 PMC6058949

[97] Adeva-Andany MM , Martinez-Rodriguez J, Gonzalez-Lucan M, Fernandez-Fernandez C, Castro-Quintela E. Insulin resistance is a cardiovascular risk factor in humans. Diabetes Metab Syndr 2019;13:1449–1455. 31336505 10.1016/j.dsx.2019.02.023

[98] Lambie M , Bonomini M, Davies SJ, Accili D, Arduini A, Zammit V. Insulin resistance in cardiovascular disease, uremia, and peritoneal dialysis. Trends Endocrinol Metab 2021;32:721–730. 34266706 10.1016/j.tem.2021.06.001 PMC8893168

[99] King GL , Park K, Li Q. Selective insulin resistance and the development of cardiovascular diseases in diabetes: the 2015 Edwin Bierman Award Lecture. Diabetes 2016;65:1462–1471. 27222390 10.2337/db16-0152 PMC4878431

[100] Viswambharan H , Yuldasheva NY, Sengupta A, Imrie H, Gage MC, Haywood N, Walker AM, Skromna A, Makova N, Galloway S, Shah P, Sukumar P, Porter KE, Grant PJ, Shah AM, Santos CXC, Li J, Beech DJ, Wheatcroft SB, Cubbon RM, Kearney MT. Selective enhancement of insulin sensitivity in the endothelium in vivo reveals a novel proatherosclerotic signaling loop. Circ Res 2017;120:784–798. 27920123 10.1161/CIRCRESAHA.116.309678

[101] Fu J , Yu MG, Li Q, Park K, King GL. Insulin's actions on vascular tissues: physiological effects and pathophysiological contributions to vascular complications of diabetes. Mol Metab 2021;52:101236. 33878400 10.1016/j.molmet.2021.101236 PMC8513152

[102] Shanik MH , Xu Y, Skrha J, Dankner R, Zick Y, Roth J. Insulin resistance and hyperinsulinemia: is hyperinsulinemia the cart or the horse? Diabetes Care 2008;31(Suppl. 2):S262–S268. 18227495 10.2337/dc08-s264

[103] Gregory JM , Cherrington AD, Moore DJ. The peripheral peril: injected insulin induces insulin insensitivity in type 1 diabetes. Diabetes 2020;69:837–847. 32312900 10.2337/dbi19-0026 PMC7171956

[104] Falciglia M , Freyberg RW, Almenoff PL, D'Alessio DA, Render ML. Hyperglycemia-related mortality in critically ill patients varies with admission diagnosis. Crit Care Med 2009;37:3001–3009. 19661802 10.1097/CCM.0b013e3181b083f7 PMC2905804

[105] Honiden S , Inzucchi SE. Metabolic management during critical illness: glycemic control in the ICU. Semin Respir Crit Care Med 2015;36:859–869. 26595046 10.1055/s-0035-1565253

[106] Wang Y , Li S, Lu J, Feng K, Huang X, Hu F, Sun M, Zou Y, Li Y, Huang W, Zhou J. Threshold of hyperglycaemia associated with mortality in critically ill patients: a multicentre, prospective, observational study using continuous glucose monitoring. Diabetologia 2024;67:1295–1303. 38568252 10.1007/s00125-024-06136-1 PMC11153265

[107] Livesey G . Health potential of polyols as sugar replacers, with emphasis on low glycaemic properties. Nutr Res Rev 2003;16:163–191. 19087388 10.1079/NRR200371

[108] DeFronzo RA , Reeves WB, Awad AS. Pathophysiology of diabetic kidney disease: impact of SGLT2 inhibitors. Nat Rev Nephrol 2021;17:319–334. 33547417 10.1038/s41581-021-00393-8

[109] Wu B , Bell K, Stanford A, Kern DM, Tunceli O, Vupputuri S, Kalsekar I, Willey V. Understanding CKD among patients with T2DM: prevalence, temporal trends, and treatment patterns-NHANES 2007–2012. BMJ Open Diabetes Res Care 2016;4:e000154. 10.1136/bmjdrc-2015-000154 PMC4838667 27110365

[110] Felicetta JV , Sowers JR. Systemic hypertension in diabetes mellitus. Am J Cardiol 1988;61:H34–H40. 10.1016/0002-9149(88)91103-4 3289349

[111] Schneider AS , Schettler A, Markowski A, Luettig B, Momma M, Seipt C, Hadem J, Wilhelmi M. Assessment of xylitol serum levels during the course of parenteral nutrition including xylitol in intensive care patients: a case control study. Clin Nutr 2014;33:483–488. 23916161 10.1016/j.clnu.2013.06.018

[112] Takayasu S , Kamba A, Yoshida K, Terui K, Watanuki Y, Ishigame N, Mizushiri S, Tomita T, Nakamura K, Yasui-Furukori N, Daimon M. Secondary oxalosis induced by xylitol concurrent with lithium-induced nephrogenic diabetes insipidus: a case report. BMC Nephrol 2020;21:157. 32357847 10.1186/s12882-020-01814-9 PMC7195762

[113] Michael M , Harvey E, Milliner DS, Frishberg Y, Sas DJ, Calle J, Copelovitch L, Penniston KL, Saland J, Somers MJG, Baum MA. Diagnosis and management of primary hyperoxalurias: best practices. Pediatr Nephrol 2024;39:3143–3155. 38753085 10.1007/s00467-024-06328-2

[114] Bonomini M , Zammit V, Divino-Filho JC, Davies SJ, Di Liberato L, Arduini A, Lambie M. The osmo-metabolic approach: a novel and tantalizing glucose-sparing strategy in peritoneal dialysis. J Nephrol 2021;34:503–519. 32767274 10.1007/s40620-020-00804-2 PMC8036224

[115] Rago C , Lombardi T, Di Fulvio G, Di Liberato L, Arduini A, Divino-Filho JC, Bonomini M. A new peritoneal dialysis solution containing L-carnitine and xylitol for patients on continuous ambulatory peritoneal dialysis: first clinical experience. Toxins (Basel) 2021;13:174. 33668249 10.3390/toxins13030174 PMC7996173

[116] Bonomini M , Davies S, Kleophas W, Lambie M, Reboldi G, Liberato LD, Divino-Filho JC, Heimburger O, Ortiz A, Povlsen J, Iacobelli M, Prosdocimi T, Arduini A. Rationale and design of ELIXIR, a randomized, controlled trial to evaluate efficacy and safety of XyloCore, a glucose-sparing solution for peritoneal dialysis. Perit Dial Int 2025;45:17–25. 39205396 10.1177/08968608241274106

[117] Gronda E , Gallieni M, Pacileo G, Capasso G, Wei LJ, Trepiccione F, Heidempergher M, Bonomini M, Zimarino M, Divino-Filho JC, Di Liberato L, Caracciolo MM, Masola V, Prosdocimi T, Iacobelli M, Vitagliano C, Arduini A. Rationale and design of PURE, a randomized controlled trial to evaluate peritoneal ultrafiltration with PolyCore in refractory congestive heart failure. Kidney Blood Press Res 2024;49: 852–862. 39197425 10.1159/000541127

[118] Balzer MS . Molecular pathways in peritoneal fibrosis. Cell Signal 2020;75:109778. 32926960 10.1016/j.cellsig.2020.109778

[119] Bartosova M , Schaefer B, Vondrak K, Sallay P, Taylan C, Cerkauskiene R, Dzierzega M, Milosevski-Lomic G, Buscher R, Zaloszyc A, Romero P, Lasitschka F, Warady BA, Schaefer F, Ujszaszi A, Schmitt CP. Peritoneal dialysis vintage and glucose exposure but not peritonitis episodes drive peritoneal membrane transformation during the first years of PD. Front Physiol 2019;10:356. 31001140 10.3389/fphys.2019.00356 PMC6455046

[120] Mor I , Cheung EC, Vousden KH. Control of glycolysis through regulation of PFK1: old friends and recent additions. Cold Spring Harb Symp Quant Biol 2011;76:211–216. 22096029 10.1101/sqb.2011.76.010868

[121] Okar DA , Manzano A, Navarro-Sabate A, Riera L, Bartrons R, Lange AJ. PFK-2/FBPase-2: maker and breaker of the essential biofactor fructose-2,6-bisphosphate. Trends Biochem Sci 2001;26:30–35. 11165514 10.1016/s0968-0004(00)01699-6

[122] Choi IY , Wu C, Okar DA, Lange AJ, Gruetter R. Elucidation of the role of fructose 2,6-bisphosphate in the regulation of glucose fluxes in mice using in vivo (13)C NMR measurements of hepatic carbohydrate metabolism. Eur J Biochem 2002;269:4418–4426. 12230553

[123] Massillon D , Chen W, Barzilai N, Prus-Wertheimer D, Hawkins M, Liu R, Taub R, Rossetti L. Carbon flux via the pentose phosphate pathway regulates the hepatic expression of the glucose-6-phosphatase and phosphoenolpyruvate carboxykinase genes in conscious rats. J Biol Chem 1998;273:228–234. 9417069 10.1074/jbc.273.1.228

[124] Bazzato G , Coli U, Landini S, Fracasso A, Morachiello P, Righetto F, Scanferla F, Onesti G. Xylitol as osmotic agent in CAPD: an alternative to glucose for uremic diabetic patients? Trans Am Soc Artif Intern Organs 1982;28:280–286. 6761936

[125] Piccapane F , Bonomini M, Castellano G, Gerbino A, Carmosino M, Svelto M, Arduini A, Procino G. A novel formulation of glucose-sparing peritoneal dialysis solutions with l-carnitine improves biocompatibility on human mesothelial cells. Int J Mol Sci 2020;22:123. 33374405 10.3390/ijms22010123 PMC7795315

[126] Masola V , Bonomini M, Onisto M, Ferraro PM, Arduini A, Gambaro G. Biological effects of XyloCore, a glucose sparing PD solution, on mesothelial cells: focus on mesothelial-mesenchymal transition, inflammation and angiogenesis. Nutrients 2021;13:2282. 34209455 10.3390/nu13072282 PMC8308380

[127] Tan Z , Wang Z, Zeng Q, Liu X, Zhang Y, Li S, Huang J, Zeng Y, Huang Z, Jin C, Fu N, Zhao Q, Mu Y, Wang Z, Xiao J, Yang H, Ke G. Natural intestinal metabolite xylitol reduces BRD4 levels to mitigate renal fibrosis. Clin Transl Sci 2024;17:e13770. 38501942 10.1111/cts.13770 PMC10949883

[128] Xu Y , Zhong Z, Li Y, Li Z, Zhou Y, Li Z, Mao H. Interaction effect between fasting plasma glucose and lipid profiles on mortality of peritoneal dialysis patients. Clin Kidney J 2023;16:727–734. 37007694 10.1093/ckj/sfac266 PMC10061421

[129] Beer JH , Allemann M. Xylitol: bitter cardiovascular data for a successful sweetener. Eur Heart J 2024;45:2453–2455. 38842099 10.1093/eurheartj/ehae252

[130] Tomonobu N , Komalasari N, Sumardika IW, Jiang F, Chen Y, Yamamoto KI, Kinoshita R, Murata H, Inoue Y, Sakaguchi M. Xylitol acts as an anticancer monosaccharide to induce selective cancer death via regulation of the glutathione level. Chem Biol Interact 2020;324:109085. 32275922 10.1016/j.cbi.2020.109085

[131] Sahasakul Y , Angkhasirisap W, Lam-Ubol A, Aursalung A, Sano D, Takada K, Trachootham D. Partial substitution of glucose with xylitol prolongs survival and suppresses cell proliferation and glycolysis of mice bearing orthotopic xenograft of oral cancer. Nutrients 2022;14:2023. 35631164 10.3390/nu14102023 PMC9148106

[132] Cannon M , Dempsey E, Cosantino A, Chandel N, Ghoreishi-Haack N. Cancer cell line inhibition by osmotic pump-administered xylitol in a syngeneic mouse model. Res Sq [Preprint]. 2024. doi:10.21203/rs.3.rs-3977059/v1

